# Role of Proinflammatory Cytokines in Feedback Modulation of Circadian Clock Gene Rhythms by Saturated Fatty Acids

**DOI:** 10.1038/s41598-019-45322-9

**Published:** 2019-06-20

**Authors:** Sam-Moon Kim, Nichole Neuendorff, David J. Earnest

**Affiliations:** 1 Department of Biology, Texas A&M University, College Station, Texas 77843-3258 USA; 2 Center for Biological Clocks Research, Texas A&M University, College Station, Texas 77843-3258 USA; 3 Department of Neuroscience and Experimental Therapeutics, Texas A & M Health Science Center, College of Medicine, Bryan, TX 77807-3260 USA

**Keywords:** Reporter genes, Circadian mechanisms

## Abstract

Proinflammatory signaling cascades have been implicated in the mechanism by which high fat diet (HFD) and saturated fatty acids (SFA) modulate fundamental circadian properties of peripheral clocks. Because the cytokines TNFα and IL-6 are key signals in HFD- and SFA-induced proinflammatory responses that ultimately lead to systemic insulin resistance, the present study examined the roles of these cytokines in the feedback modulation of peripheral circadian clocks by the proinflammatory SFA, palmitate. IL-6 and TNFα secretion in *Bmal1-dLuc* fibroblast cultures was increased during palmitate treatment although the time course and amplitude of the inductive response differed between these cytokines. Similar to the time-dependent phase shifts observed in response to palmitate, treatment with IL-6 or with the low dose (0.1 ng/ml) of TNFα at hour 12 (i.e., after forskolin synchronization) induced phase advances of fibroblast *Bmal1-dLuc* rhythms. In complementary experiments, treatment with neutralizing antibodies against these proinflammatory cytokines or their receptors to inhibit of IL-6- or TNFα-mediated signaling repressed palmitate-induced phase shifts of the fibroblast clock. These studies suggest that TNFα, IL-6 and other proinflammatory cytokines may mediate the feedback modulation of peripheral circadian clocks by SFA-induced inflammatory signaling.

## Introduction

Circadian clocks in the suprachiasmatic nucleus (SCN) and peripheral tissues mediate the generation and entrainment of tissue- and cell-specific rhythms in various physiological processes such as inflammation and metabolism^1^. While light-dark cues are the primary signals for circadian entrainment in mammals, nutrient metabolism and food intake, especially high fat diet (HFD), can modulate the circadian timekeeping function of SCN and peripheral clocks. HFD has been shown to alter the period of the activity rhythm and clock gene rhythms in peripheral tissues^2,3^. In addition, recent findings indicate that HFD and the proinflammatory saturated fatty acid (SFA) palmitate, which is a major constituent of HFD, disrupt the diurnal oscillations of glucagon-like peptide 1 and insulin secretion and damp clock gene rhythms in intestinal L-cells^4^. Our studies have yielded similar effects of HFD and palmitate on circadian clocks *in vivo* and *in vitro*; HFD feeding lengthened the period of the activity rhythm and peripheral clock gene rhythms^3^, and prolonged (48 hr) or acute (4 hr) treatment with palmitate respectively induced increases in the period or time-dependent shifts in the phase of fibroblast *Bmal1-dLuc* rhythms *in vitro*^5^. In view of increasing evidence for the association between circadian dysregulation and metabolic phenotypes, this diet-mediated modulation of circadian clock function may contribute to the mechanism by which HFD and SFAs induce systemic metabolic disorders.

Chronic inflammation in peripheral tissues is a critical factor in diet-induced metabolic disorders including obesity and insulin resistance. In diet-related metabolic dysfunction, HFD and SFAs trigger proinflammatory responses in various peripheral tissues through the induction of NF-κB and JNK activation and increased secretion of proinflammatory cytokines such as IL-6 and TNFα^6^, ultimately leading to systemic insulin resistance^7–9^. Because the modulatory effects of HFD and SFAs on metabolism and circadian timekeeping are closely linked, proinflammatory signaling cascades may function as a common mediator of diet-induced metabolic and circadian dysregulation. This speculation is indirectly supported by our previous findings that in palmitate-treated fibroblasts *in vitro* and in HFD-fed mice, the resultant increases in inflammatory signaling through the induction of NF-κB and JNK activity and IL-6 expression occur concurrently with corresponding alterations of the circadian period or phase of core clock gene rhythms^3,5^. Furthermore, DHA and other inhibitors of inflammatory signaling such as AICAR and cardamonin attenuate palmitate-induced proinflammatory responses and phase shifts of clock gene rhythms in cultured fibroblasts^5^. Furthermore, HFD has been shown to disrupt circadian rhythms of PPARγ and AMPK^10,11^, which are directly involved in the regulation of metabolism and may also link the clock machinery to mediators of inflammation. Collectively, these observations raise the possibility that key signaling cascades mediating diet-related inflammation in peripheral tissues may also govern the modulation of circadian clock function by HFD and SFAs.

The immune system is clock-controlled, but also feeds back to regulate the timekeeping function of circadian clocks. The modulatory effects of the immune system on circadian timekeeping and the underlying clock mechanism have been observed in studies demonstrating that lipopolysaccharide (LPS) administration induces phase shifts of the activity rhythm in mice^12^ and represses SCN expression of the clock genes, *Per2* and *Dbp*^13^. Alterations in the functional properties of SCN and peripheral circadian clocks induced by LPS and other inflammatory stimuli such as HFD and SFAs are presumably mediated through the release of proinflammatory cytokines. Similar to LPS treatment, intracerebroventricular injection of TNFα or IL-1β at CT15 induces phase delays of the circadian rhythm in locomotor activity and conversely, antagonism with injection of soluble TNFα receptor attenuates LPS-induced phase delays^14^. Because our previous studies indicate that time-dependent phase shifts of fibroblast circadian clocks in response to palmitate are associated with corresponding increases in IL-6 expression^5^, it is possible that the modulation of circadian phase and period following palmitate treatment is derived from SFA-induced feedback on the clock mechanism via inflammatory signaling through proinflammatory cytokines. To test this hypothesis, we examined the role of the proinflammatory cytokines, IL-6 and TNFα, in mediating palmitate-induced phase shifts of the fibroblast clock using two different approaches. Experiments first determined whether the proinflammatory cytokines, IL-6 and TNFα, mimic the phase shifting effects of palmitate at hour 12 when this SFA induces maximal phase shifts in *Bmal1-dLuc* fibroblasts. In subsequent studies, neutralizing antibodies against these proinflammatory cytokines or their receptors were used to conversely determine whether inhibition of IL-6- or TNFα-mediated signaling abates palmitate-induced phase shifts.

## Results

### Effects of palmitate on fibroblast IL-6 and TNFα secretion

**Figure 1:**
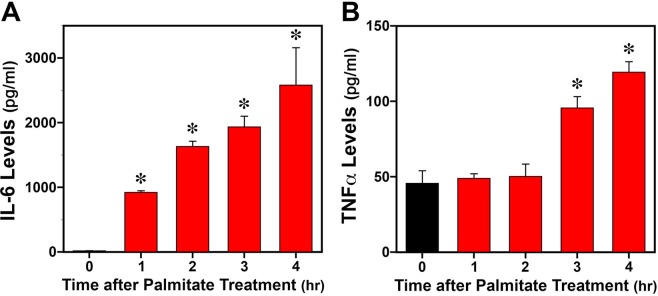
Effects of acute palmitate (PAL) treatment on IL-6 and TNFα secretion in *Bmal1-dLuc* fibroblast cultures. Bar graphs depict ELISA analysis of IL-6 (**A**) and TNFα (**B**) levels in culture medium collected from *Bmal1-dLuc* fibroblasts at 0, 1, 2, 3 and 4 hr after palmitate (250 μM) administration for 4 hr at hour 12. Asterisks indicate times at which palmitate-induced IL-6 or TNFα secretion in fibroblast cultures was significantly increased (p < 0.05) in comparison with levels observed prior to treatment (at 0 hr).

Inflammatory signaling through increased secretion of proinflammatory cytokines is a critical process in the mechanism by which SFAs such as palmitate mediate metabolic dysregulation. In this regard, palmitate has been shown to induce signaling cascades characterized by increased secretion of proinflammatory cytokines^15^. To explore the role of the proinflammatory cytokines in the mechanism by which palmitate phase shifts the fibroblast clock, we first determined whether this SFA similarly induces IL-6 and TNFα secretion in *Bmal1-dLuc* fibroblasts. Prior to palmitate treatment, Il-6 levels in the culture medium were consistently low (8–19 pg/ml) and near assay limits of detection whereas basal levels of TNFα secretion were higher with concentrations in the medium ranging from 30–60 pg/ml (Fig. 1). Palmitate treatment had significant effects (p < 0.05) in inducing both IL-6 and TNFα secretion in *Bmal1-dLuc* fibroblasts although the kinetics and amplitude of the response differed between these proinflammatory cytokines. Palmitate-induced IL-6 secretion occurred rapidly beginning 1 hour after treatment and levels in the medium were increased by 60–170-fold relative to basal concentrations. In contrast, the effect of palmitate on fibroblast TNFα secretion was delayed such that increased levels were only observed at 3 and 4 hours after treatment, and the amplitude of the response was lower (2–2.6-fold increase).

### Phase shifting effects of recombinant IL-6 and TNFα on fibroblast clock gene rhythms

**Figure 2:**
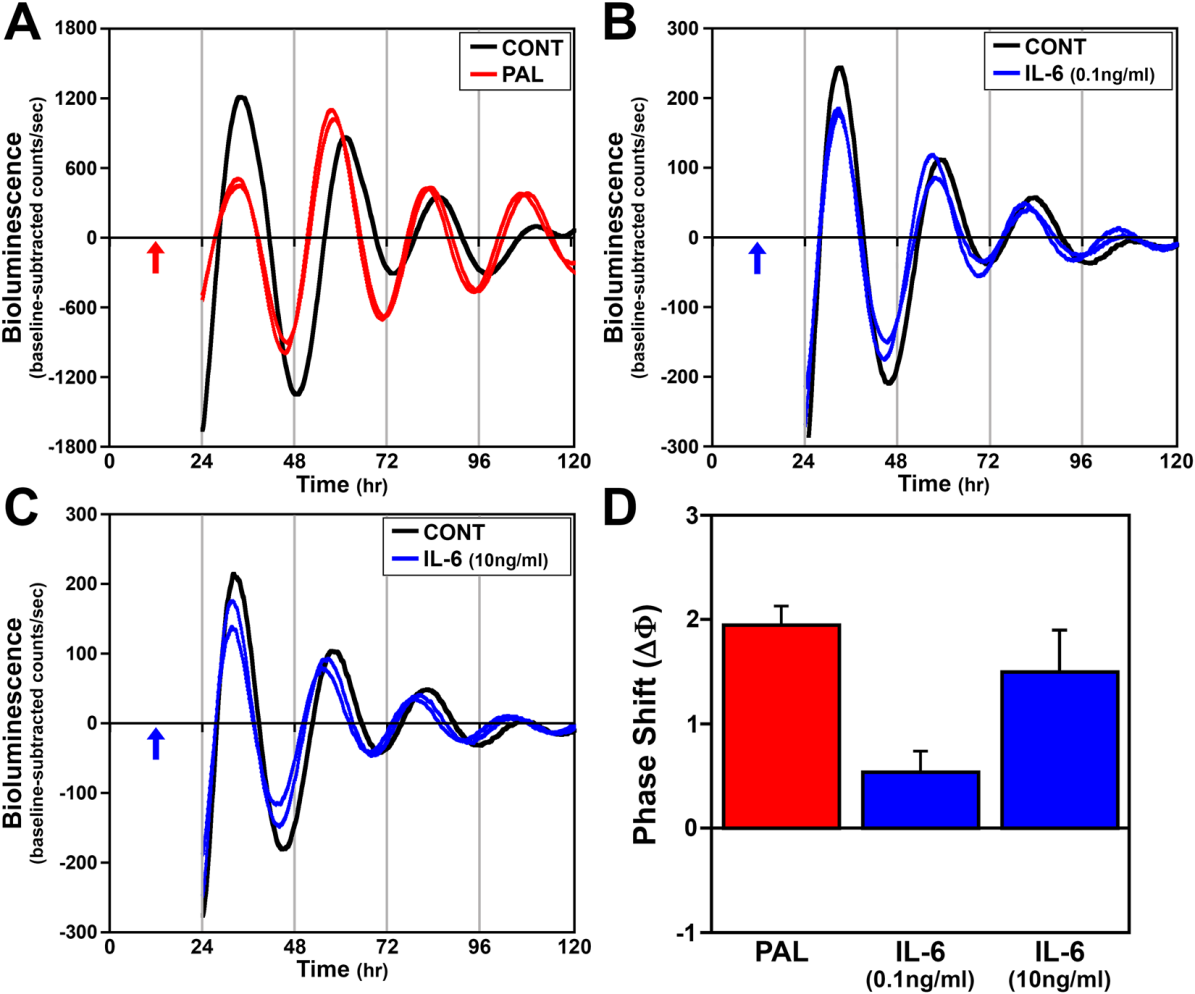
Phase-shifting effects of acute IL-6 treatment on ensemble *Bmal1-dLuc* rhythms in cultured fibroblasts. Representative recordings of ensemble bioluminescence from two individual cultures of *Bmal1-dLuc* fibroblasts treated for 4 hr with (**A**) 250 µM palmitate (**PAL**), (**B**) 0.1 ng/ml or (**C**) 10 ng/ml recombinant **IL-6** at hour 12 in comparison with an experiment-matched PBS control (**CONT**). Arrows indicate the time of PBS, PAL or IL-6 treatment. (**D**) Amplitude of phase shifts (∆Φ) of fibroblast *Bmal1-dLuc* rhythms in response to treatment with PAL (n = 6), 0.1 ng/ml IL-6 (n = 8) or 10 ng/ml IL-6 (n = 8) for 4 hr at hour 12. Bar graph depicts mean ( ± SEM) phase shifts (ΔΦ) in hours as a function of treatment group.

**Figure 3:**
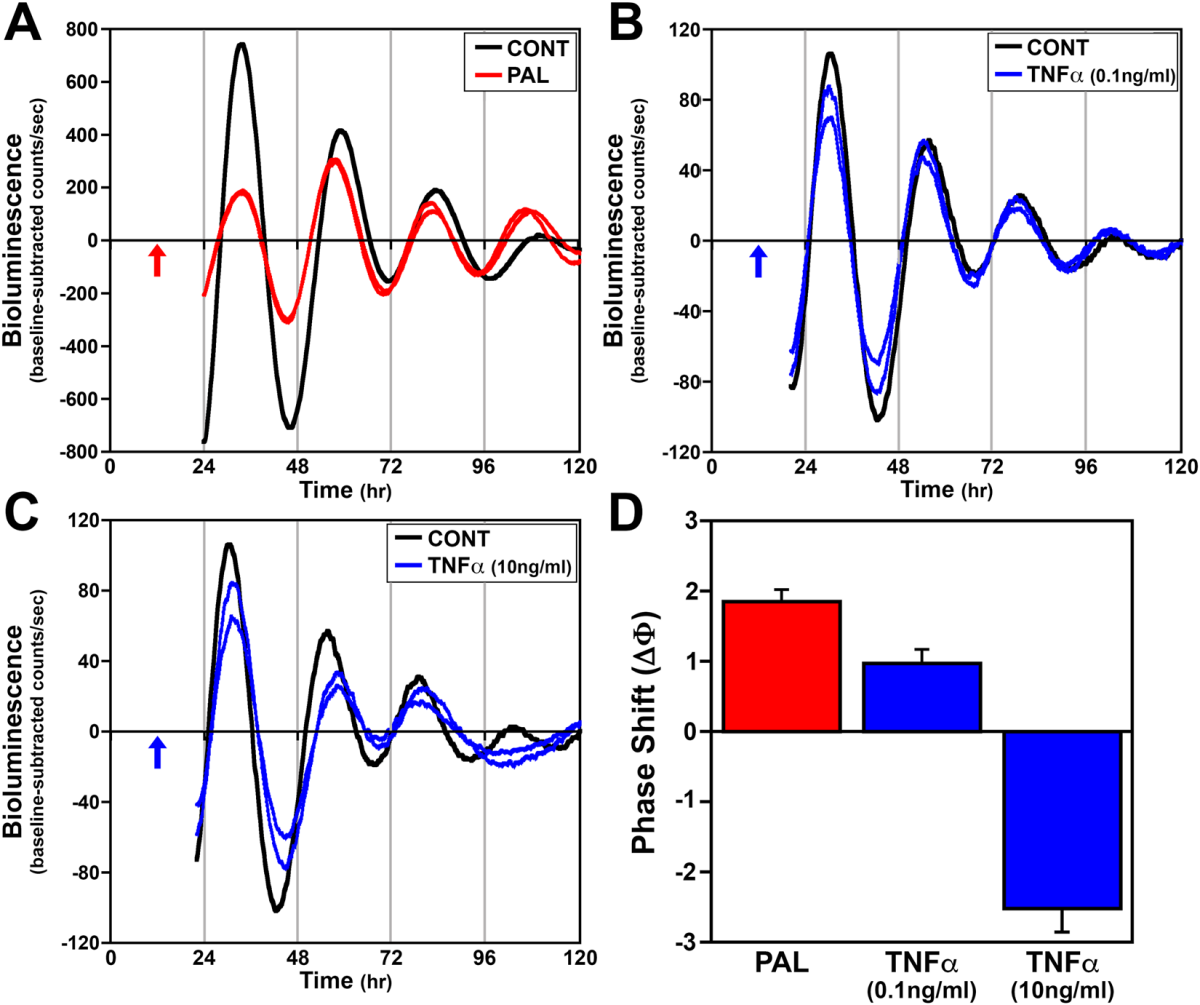
Phase-shifting effects of acute TNFα treatment on ensemble *Bmal1-dLuc* rhythms in cultured fibroblasts. Representative recordings of ensemble bioluminescence from two individual cultures of *Bmal1-dLuc* fibroblasts treated for 4 hr with (**A**) 250 µM palmitate (**PAL**), (**B**) 0.1 ng/ml or (**C**) 10 ng/ml recombinant **TNFα** at hour 12 in comparison with an experiment-matched PBS control (**CONT**). Arrows indicate the time of PBS, PAL or TNFα treatment. (**D**) Amplitude of phase shifts (∆Φ) of fibroblast *Bmal1-dLuc* rhythms in response to treatment with PAL (n = 6), 0.1 ng/ml TNFα (n = 8) or 10 ng/ml TNFα (n = 8) for 4 hr at hour 12. Bar graph depicts mean ( ± SEM) phase shifts (∆Φ) in hours as a function of treatment group. Phase advances of the fibroblast *Bmal1-dLuc* rhythm are indicated by positive values and delays are denoted by negative values.

Our previous studies indicate that proinflammatory and phase-shifting effects of palmitate are time-dependent and contemporaneous, such that this SFA coincidentally induces peak phase shifts of clock gene rhythms, NF-κB activation and IL-6 expression at hour 12 in fibroblasts^5^. Thus, we next determined whether treatment with recombinant IL-6 at hour 12 mimics the phase shifting effects of palmitate on fibroblast *Bmal1-dLuc* rhythms. Following to acute (4 hr) treatment with 0.1 ng/ml or 10 ng/ml IL-6 at hour 12, the period of fibroblast *Bmal1-dLuc* rhythms (24.3 ± 0.2 hr and 24.1 ± 0.5 hr, respectively) was not significantly different from that observed in experiment-matched PBS controls (24.6 ± 0.1 hr). However, 0.1 ng/ml and 10 ng/ml IL-6 significantly decreased (p < 0.05) the amplitude of fibroblast *Bmal1-dLuc* rhythms (654.8 ± 20.8; 588.3 ± 32.5) relative to that observed in PBS controls (752.8 ± 32.3). Phase shifting analysis revealed that the effects of IL-6 treatment at hour 12 on fibroblast *Bmal1-dLuc* rhythms were dose-dependent; treatment with 10 ng/ml IL-6 generated large phase advances of 1.5 hours that were comparable in amplitude to the phase shifts in response to palmitate treatment (Fig. 2) whereas the phase advances induced by the 0.1 ng/ml dose of this proinflammatory cytokine were much smaller (≈0.5 hr). Because increased secretion of TNFα is also associated with inflammatory signaling cascades induced by HFD and SFAs^15,16^, we next tested whether this proinflammatory cytokine also induces phase shifts of fibroblast *Bmal1-dLuc* rhythms. In response to treatment at hour 12, 0.1 ng/ml TNFα had no effect (24.6 ± 0.1 hr) whereas the 10 ng/ml dose significantly increased (p < 0.05) the period of fibroblast *Bmal1-dLuc* rhythms (26.2 ± 0.3 hr) relative to that observed in PBS controls (24.7 ± 0.1 hr). The amplitude of *Bmal1-dLuc* rhythms in fibroblasts treated with TNFα (0.1 ng/ml: 167.3 ± 7.4; 10 ng/ml: 171.7 ± 10.7) was significantly decreased (p < 0.05) relative to that observed in PBS controls (243.7 ± 11.8). In contrast to the specific directionality of the shifts induced by palmitate and IL-6, TNFα treatment at hour 12 produced both phase advances and delays of fibroblast *Bmal1-dLuc* rhythms. The direction of these TNFα-induced shifts was dose-dependent such that phase advances of approximately 1 hr were observed following acute treatment with the low dose (0.1 ng/ml) of this proinflammatory cytokine whereas the high dose (10 ng/ml) induced large phase delays of ≈2.5 hr (Fig. 3).

### Effect of IL-6 receptor α and TNFα neutralizing antibodies on palmitate-induced phase shifts of fibroblast clock gene rhythms

**Figure 4:**
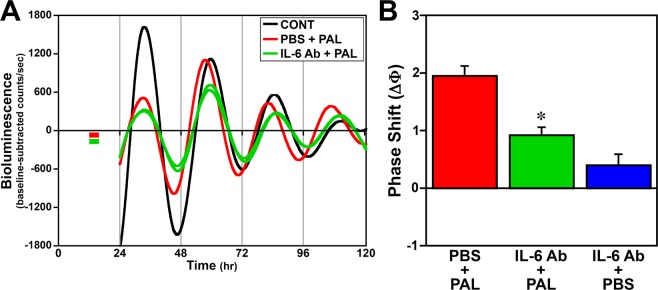
Inhibitory effects of treatment with neutralizing antibody against IL-6 receptor α on the phase-shifting responses of *Bmal1-dLuc* fibroblasts to palmitate. (**A**) Representative recordings of ensemble bioluminescence from individual cultures of *Bmal1-dLuc* fibroblasts treated at hour 12 for 4 hr with vehicle (PBS/BSA, CONT, n = 8), 250 µM palmitate (PBS + PAL), or neutralizing antibody to IL-6 receptor α (0.78 µg/ml) in conjunction with palmitate administration (IL-6Rα Ab + PAL). Colored bars denote time of treatment with IL-6 neutralizing antibody (green) and/or palmitate (red). (**B**) Amplitude of phase shifts (∆Φ) of fibroblast *Bmal1-dLuc* rhythms in response to treatment with PAL (n = 8), IL-6Rα Ab + PAL (n = 8) or neutralizing antibody treatment alone (IL-6Rα Ab + PBS, n = 8) for 4 hr at hour 12. Bar graph depicts the mean ( ± SEM) phase shifts (ΔΦ) in hours as a function of treatment group. Asterisks indicate that phase shifts of the *Bmal1-dLuc* rhythm were significantly decreased (p < 0.05) in fibroblasts treated with neutralizing antibody to IL-6 receptor α (IL-6 Ab + PAL) in comparison with those observed in response to palmitate alone (PBS + PAL).

**Figure 5:**
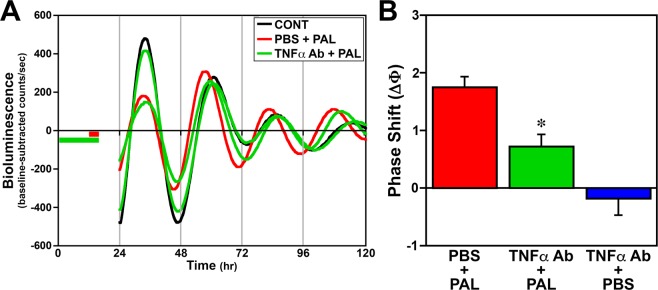
Inhibitory effects of treatment with neutralizing antibody against TNFα on the phase-shifting responses of *Bmal1-dLuc* fibroblasts to palmitate. Representative recordings of ensemble bioluminescence from individual cultures of *Bmal1-dLuc* fibroblasts treated at hour 12 for 4 hr with vehicle (PBS/BSA, CONT, n = 8), 250 µM palmitate (PBS + PAL), or neutralizing antibody to TNFα (0.4ug/ml) in conjunction with palmitate administration (TNFα Ab + PAL). Colored bars denote time of palmitate exposure (red) after 12 hr pretreatment with TNFα neutralizing antibody (green). (B) Amplitude of phase shifts (∆Φ) of fibroblast *Bmal1-dLuc* rhythms in response to treatment with PAL (n = 8), TNFα Ab + PAL (n = 8) or neutralizing antibody treatment alone (TNFα Ab + PBS, n = 8) for 4 hr at hour 12. Bar graph depicts the mean (±SEM) phase shifts (ΔΦ) in hours as a function of treatment group. Asterisks indicate that phase shifts of the *Bmal1-dLuc* rhythm were significantly decreased (p < 0.05) in *Bmal1-dLuc* fibroblasts treated with neutralizing antibody to TNFα (TNFα Ab + PBS) in comparison with those observed in response to palmitate alone (PBS + PAL).

To complement our analysis of the phase-shifting responses to proinflammatory cytokines, we subsequently applied the converse approach using neutralizing antibodies against IL-6 receptor α or TNFα to determine whether antagonism of cytokine-mediated inflammatory signaling inhibits palmitate-induced phase shifts. In comparison with experiment-matched PBS controls, treatment with neutralizing antibody to IL-6 receptor α or to TNFα alone or in conjunction with palmitate exposure had no significant effect on the period of fibroblast *Bmal1-dLuc* rhythms (**IL-6Rα**: PBS = 25.5 ± 0.4 hr; IL-6Rα Ab + PAL = 25.2 ± 0.1 hr; IL-6Rα Ab = 25.4 ± 0.1 hr; **TNFα**: PBS = 25.7 ± 0.4 hr; TNFα Ab + PAL = 25.7 ± 0.1 hr; TNFα Ab = 25.9 ± 0.3 hr). The phase-shifting effects of palmitate at hour 12 on fibroblast *Bmal1-dLuc* rhythms were abated by concurrent treatment with neutralizing antibody to IL-6 receptor α. In cultures treated with IL-6 receptor α neutralizing antibody, phase shifting responses of the *Bmal1-dLuc* rhythm to palmitate were significantly decreased (p < 0.05) relative to those observed in palmitate-treated controls (Fig. 4). Administration of palmitate alone induced 2-hour phase advances in PBS-treated controls whereas the amplitude of these shifts was reduced approximately by 53% in cultures treated with IL-6 receptor α neutralizing antibody during exposure to palmitate at hour 12. Treatment with neutralizing antibody alone had little or no phase-shifting effect on the *Bmal1-dLuc* rhythm. Similar to the effects of IL-6 receptor α antagonism, treatment with neutralizing antibody to TNFα for 12 hr in advance and during exposure to palmitate at hour 12 significantly decreased (p < 0.05) the phase-shifting effects of this SFA on fibroblast *Bmal1-dLuc* rhythms (Fig. 5). As observed previously, palmitate alone induced large phase advances of 1.8 hr in PBS-pretreated controls. In comparison, the amplitude of these palmitate-induced phase shifts was abated by ≈58% in *Bmal1-dLuc* fibroblasts pre-treated with TNFα neutralizing antibody. The phase-shifting effects of treatment with TNFα neutralizing antibody alone were negligible.

## Discussion

HFD and SFAs modulate the timekeeping function of peripheral circadian clocks in mammals. Long-term and short-term HFD treatment has been shown to alter circadian properties (i.e., decrease amplitude, increase period, phase shift) of clock gene rhythms in peripheral tissues^2,3,17^. Several investigations, including our recent study, have also demonstrated that the proinflammatory SFA, palmitate, similarly modulates circadian clock properties in fibroblasts, liver and hypothalamic cells^5,18,19^. Increasing evidence indicates that inflammatory signaling may play a critical role in the palmitate-induced feedback modulation of circadian timekeeping in peripheral clocks. In this regard, palmitate induces time-dependent increases in NF-κB activation and IL-6 expression that coincide with its peak phase-shifting effects and various inhibitors of inflammatory signaling repress the proinflammatory and phase-shifting responses to this proinflammatory SFA^5^. The present data unveil the potential roles of proinflammatory cytokines in the feedback modulation of peripheral circadian clocks by HFD- and SFA-mediated inflammation; we found that treatment with recombinant IL-6 or with the low dose of TNFα mimics the phase-shifting effects of palmitate on fibroblast circadian clocks whereas administration of neutralizing antibodies against IL-6 receptor α or TNFα attenuate phase-shifting responses to this SFA. Implications for TNFα and IL-6 in the pathway by which inflammation modulates circadian timekeeping in peripheral clocks are reinforced by studies demonstrating that: 1) TNFα alters liver and fibroblast clock gene expression;^20^ 2) TNFα induces phase shifts in the circadian rhythm of locomotor activity *in vivo*;^14^ 3) LPS-induced phase shifts of the activity rhythm are blocked following administration of soluble TNFα receptor^14^ and in TNFα receptor-deficient (1/p55, Tnfr1) mice;^21^ and 4) IL-6-deficient mice are characterized by changes in hippocampal clock gene expression and the temporal architecture of the activity rhythm^22^. Collectively, these observations suggest that TNFα, IL-6 and other proinflammatory cytokines may be key elements in the signaling cascade by which HFD- and SFA-induced inflammation feeds back and modulates fundamental circadian properties of peripheral clocks.

Although our current results demonstrate that the proinflammatory cytokines IL-6 and TNFα shift the phase of fibroblast *Bmal1-dLuc* rhythms in a time dependent manner, it is unclear why the directionality of phase shifts in response to the high dose of TNFα was different from those induced by the low dose of this cytokine and by treatment with IL-6 or palmitate alone. One possible explanation for the induction of phase delays, rather than advances, in response to 10 ng/ml TNFα is that this effect is pharmacological because the dose is much higher than the physiological levels observed in our experiments and other studies where 250 µM palmitate increased the concentration of TNFα in the culture medium to ≈0.05–0.12 ng/ml^8^. Consistent with this explanation, the high dose of TNFα produced phase delays that were associated with large increases (≈1.5 hr) in the period of fibroblast *Bmal1-dLuc* rhythms whereas all other treatments including the low dose of TNFα (0.1 ng/ml), which is equivalent to the physiological levels observed in response to palmitate administration, induced phase advances in the absence of any significant alterations in period. Furthermore, similar pharmacological effects of the high dose were evident even when TNFα treatment occurred at other circadian times, as the resulting phase delays were consistently a function of dose-mediated increases in the period of fibroblast *Bmal1-dLuc* rhythms.

The specific mechanism by which palmitate-induced inflammatory signaling and downstream induction of proinflammatory cytokines like TNFα and IL-6 mediate the feedback modulation of peripheral circadian clocks is unknown. Recent evidence suggests that key mediators of HFD- and SFA-induced inflammation may directly interact with core clock components and modulate clock gene expression. Activation of the transcription factor NF-κB mediates the inductive effects of palmitate and other SFAs on proinflammatory cytokine secretion by various peripheral tissues^7–9^. In addition to mediating SFA-induced secretion of proinflammatory cytokines, NF-κB has been shown to interact with and regulate core clock components. The regulatory NF-κB subunit, RelB, physically interacts with BMAL1 at the chromatin level and represses CLOCK/BMAL1-mediated circadian transcription in fibroblasts^23^. Furthermore, NF-κB also forms protein complexes with CLOCK that up-regulate NF-κB–mediated transcriptional activation^24^. Based on these observations, palmitate-induced NF-κB activity may lead to increased binding with BMAL1 or CLOCK, thereby sequestering available binding partners and inhibiting the formation of BMAL1-CLOCK heterodimers. Consistent with the reported interactions between NF-κB and core clock components, our previous studies demonstrate that treatment with the NF-ĸB inhibitor, cardamonin, represses palmitate-induced proinflammatory cytokine expression and phase shifts of the fibroblast clock^5^. Similar to NF-κB, the downstream induction of the proinflammatory cytokines TNFα and IL-6 in response to palmitate may mediate inflammatory feedback between the immune system and circadian clocks via direct interactions with and/or regulation of core components of the clock mechanism in peripheral tissues. Evidence for the role of these proinflammatory cytokines in the regulation of the core clock genes is derived from studies indicating that TNFα suppresses *Per1*-*3* and clock-controlled gene expression by direct inhibition of CLOCK/BMAL1-induced E-box transactivation in fibroblasts^20^, and that IL-6 stimulates *Per1* gene expression in hepatoma cells^25^. Because many inflammatory processes including NF-κB activation^26^ and cytokine release^27^ are clock-controlled, the present findings suggest that TNFα, IL-6 and perhaps other proinflammatory cytokines are key signals that mediate mutual feedback interactions between SFA-induced inflammation and modulation of peripheral circadian clocks. Thus, future studies using chronotherapeutic strategies for treatment with the TNF-𝛼 inhibitor etanercept or anti-TNF antibody Infliximab are warranted to examine their efficacy in the management of metabolic pathophysiology associated with HFD and/or shift work schedules.

## Materials and Methods

### Cell culture

*Bmal1-dLuc* fibroblasts (Dr. Andrew Liu, University of Memphis, Memphis, TN^28^) were propagated on 60 mm dishes in Dulbeco’s Modified Eagle Medium (DMEM; HyClone) containing 10% Fetal Bovine Serum (FBS), 292 µg/ml L-glutamine, 100 units/ml penicillin, and 100 µg/ml streptomycin and maintained at 37 °C and 5% CO_2_. Medium was replaced every 48 hr and cultures were split 1:4 every 3 days.

### Fatty acid/drug preparation and treatment

Palmitate (Sigma) was dissolved in ethanol and then diluted (1:5.4 ratio) with 10% BSA (fatty acid-free and low endotoxin) diluted in 0.1 M phosphate-buffered saline (PBS). Palmitate treatment in these studies was based on physiological concentrations that have been previously observed *in vivo* or used for *in vitro* studies^5,8,29–31^.

IL-6 and TNFα recombinant proteins (Invitrogen) were dissolved in 0.1 M PBS, and then diluted (IL-6 at 1:100000 and 1:1000; TNFα at 1:200000 and 1:2000) in culture medium to achieve final concentrations of 0.1 ng/ml and 10 ng/ml, respectively. To neutralize the effects of these proinflammatory cytokines, antibodies (R&D Systems) against mouse IL-6 receptor α (AF 1830) and mouse TNFα (AF 410) were dissolved in 0.1 M PBS, and respectively diluted 1:100 or 1:1000 in culture medium to achieve final concentrations of 0.78ug/ml and 0.4ug/ml. The IL-6 receptor α antibody neutralizes IL-6-stimulated proliferation of T1165.85.2.1 mouse plasmacytoma cells and inhibition of IL-6 signaling by treatment with a similar IL-6 receptor antibody has been shown to suppress inflammation-associated colorectal cancer in diet-induced obesity^32^. The TNFα antibody neutralizes TNFα-stimulated cytotoxicity in L-929 mouse fibroblasts and prevents TNFα-mediated recurrence of B16tk tumors *in vivo*^33^. Similarly, another neutralizing antibody against TNFα has been shown to inhibit TNFα-induced NFκB activation^34^. Vehicle controls for recombinant proteins and antibodies were treated with PBS and controls for fatty acid treatment were exposed to BSA diluted in PBS with an equivalent ratio of ethanol.

### Effects of palmitate on fibroblast IL-6 and TNFα secretion

*Bmal1-dLuc* fibroblasts cultured on 35 mm dishes in DMEM medium supplemented with 25 mM HEPES, 292 µg/ml L-glutamine, 100units/ml penicillin and 100 μg/ml streptomycin were exposed for 2 hr to medium containing 15uM forskolin (hour 0) to facilitate circadian oscillation synchronization across cultures^35^ and then treated with 250 µM palmitate for 4 hr at hour 12. IL-6 and TNFα levels were analyzed in culture medium (200 µl) collected at 0, 1, 2, 3 and 4hrs after treatment. Following centrifugation to remove cell debris, concentrations of these cytokines in culture medium supernatants were determined by ELISA (ab222503 and ab208348; AbCam), according to manufacturer’s instructions. Briefly, standards, controls (stock medium containing palmitate), and duplicate aliquots of culture medium (50 µl) were loaded into 96-well microplate strips with IL-6 or TNFα antibody cocktail and incubated at room temperature for 1 hour. With intervening washes, plates were incubated with 100 µl of tetramethylbenzidine (TMB) substrate solution for 10–20 minutes in the dark. The color reaction was stopped by an equal volume of stop solution and read at 450 nm in a microplate reader (Bio-Tek). Standard curves were established from optical densities of wells containing known dilutions of standard (TNFα: 24.44–3000 pg/ml; IL-6: 7.8–1000 pg/ml) using KC3 software (Bio-Tek), and sample measurements were interpolated from standard curves. IL-6 and TNFα were undetectable in stock palmitate-containing DMEM medium. The intra-assay coefficients of variation were 5.4% (TNFα) and 3.6% (IL-6).

### Phase shifting effects of recombinant IL-6 and TNFα on fibroblast clock gene rhythms

Based on findings from the preceding experiment that palmitate induces fibroblast IL-6 and TNFα secretion, we next determined whether these proinflammatory cytokines phase shift clock gene rhythms similar to palmitate. Cultures of *Bmal1-dLuc* fibroblasts on 35 mm dishes were exposed for 2 hr to medium containing 15uM forskolin (hour 0) to facilitate circadian oscillation synchronization across cultures^35^ and then treated for 4 hr with IL-6 or TNFα recombinant proteins (final concentrations = 0.1 ng/ml or 10 ng/ml) at hour 12 when the phase shifting effects of palmitate are maximal^5^. Phase-shifting effects of IL-6 and TNFα treatment were compared relative to fibroblast cultures treated with 250 µM palmitate for 4 hr at hour 12. Vehicle controls for IL-6 and TNFα recombinant protein treatments consisted of cultures in which an equivalent amount of PBS was added to the medium. After treatment, all control and cytokine-treated cultures were placed in recording media for bioluminescence analysis of phase shifting effects on *Bmal1-dLuc* oscillations.

### Effect of IL-6 receptor α and TNFα neutralizing antibodies on palmitate-induced phase shifts of fibroblast clock gene rhythms

Neutralizing antibodies against IL-6 receptor α or TNFα were used to test whether inhibition of inflammatory signaling via these cytokines blocks or attenuates phase shifting effects of palmitate at hour 12 when this SFA induces maximal advances of the *Bmal1-dLuc* rhythm^5^. Peak phase-shifting responses were examined in *Bmal1-dLuc* fibroblasts that were treated with: 1) palmitate (250 µM) alone at hour 12 for 4 hr; 2) IL-6 receptor α neutralizing antibody (0.78 µg/ml) for 4 hr in conjunction with palmitate administration at hour 12; 3) TNFα neutralizing antibody (0.4 µg/ml) for 12 hr prior to palmitate administration at hour 12 for 4 hr; and 4) IL-6 receptor α (for 4 hr) or TNFα (for 16 hr) neutralizing antibody alone. The antibody concentrations and specific treatment protocols used in these experiments are based on pilot studies; lower doses of these neutralizing antibodies or treatment with TNFα neutralizing antibody for shorter duration (i.e., 4 hr in only conjunction with palmitate administration) were found to have negligible effects on phase-shifting effects of palmitate. The vehicle controls for IL-6 receptor α and TNFα neutralizing antibody treatments consisted of cultures in which an equivalent amount of PBS was added to the medium. Following treatment, cultures were placed in recording media for bioluminescence analysis of treatment effects on palmitate-induced phase shifts of *Bmal1-dLuc* oscillations.

### Real-time analysis of Bmal1-dLuc rhythms

Prior to bioluminescence analysis of *Bmal1-dLuc* fibroblast cultures on 35 mm dishes, growth medium containing control or experimental treatments (IL-6 or TNFα recombinant proteins, neutralizing antibodies, palmitate, or BSA) was removed. Cultures were rinsed, placed in DMEM recording medium containing 15uM forskolin, 25 mM HEPES, 292 µg/ml L-glutamine, 100units/ml penicillin, 100 μg/ml streptomycin and 10uM luciferin (Promega) and then sealed airtight with sterile glass coverslips and sterile silicon grease. The temporal patterns of *Bmal1-dLuc* bioluminescence were analyzed using an automated 32-channel luminometer (LumiCycle; Actimetrics) housed in a standard culture incubator at 35 °C. Bioluminescence from individual cultures was continuously recorded for ~70 sec at intervals of 10 min and analyzed using the Lumicycle Analysis program. As described previously^5,36^, rhythm parameters (period, amplitude) were determined from baseline-subtracted data using the damped sine fit and Levenberg–Marquardt algorithm (Y(t) = A*sin (2πft + ϕ)*e^−t/τ^ + C). The amplitude of phase shifts in response to treatment with IL-6 or TNFα recombinant proteins, or palmitate was determined by measuring the time difference between the peaks of the *Bmal1-dLuc* rhythms during the third cycle in PBS, BSA or BSA/vehicle (PBS) controls and experimental treatment groups.

### Statistical analysis

Independent *t*-tests were performed to: 1) assess the effects of palmitate on fibroblast IL-6 and TNFα secretion; 2) compare the phase-shifting effects of recombinant IL-6 or TNFα treatment relative to palmitate-induced phase shifts; and 3) determine the significance of neutralizing antibody (IL-6 receptor α or TNFα) effects on peak phase-shifting responses to palmitate. In each case, the α-value was set at 0.05.
